# Interaction between neuromuscular junction metabolic requirements in fragile X syndrome and glycogen storage disease models

**DOI:** 10.1242/dmm.052183

**Published:** 2025-09-01

**Authors:** Aashi Gurijala, Emma Rushton, Shannon N. Leahy, Nichalas Nelson, Charles R. Tessier, Kendal Broadie

**Affiliations:** ^1^ Department of Biological Sciences, Vanderbilt University, Nashville, TN 37235, USA; ^2^ Department of Medical and Molecular Genetics, Indiana University School of Medicine, Indianapolis, IN 46202, USA; ^3^ Kennedy Center for Research on Human Development, Vanderbilt University Medical Center, Nashville, TN 37235, USA

**Keywords:** Fragile X ribonucleoprotein 1, Phosphorylase kinase regulatory subunit alpha 2, Fat body protein 1, Synapse, *Drosophila*

## Abstract

A classic human patient comorbidity of fragile X syndrome and glycogen storage disease type IX has symptoms far more severe than those for either disease alone. Causal variants result in loss of the translational regulator fragile X ribonucleoprotein 1 (FMRP) and metabolic regulator phosphorylase kinase regulatory subunit alpha 2 (PHKA2), respectively. We hypothesized FMRP-PHKA2 interaction based on unsustainably elevated metabolic demand. In *Drosophila* disease models, single null mutants were viable, but double knockout (DKO) animals exhibited 100% synthetic lethality, showing an essential interaction. Metabolically, *dFMRP* and *dPHKA2* loss alone caused opposing changes in glycogen and fat stores, but DKO animals had both energy stores returned to normal. Regulatory Fat body protein 1 was elevated in single disease models but likewise returned to normal in the DKO animals. In tests of neurological phenotypes, neuromuscular junction mitochondrial function, synapse architecture and neurotransmission strength were all elevated by *dFMRP* loss, but these synaptic properties were restored to normal levels by co-removal of *dPHKA2* in DKO animals. Thus, *dFMRP* and *dPHKA2* strongly interact in metabolic and neuromuscular mechanisms, without explaining the DKO lethal interaction.

## Teaser

**Summary:** Combined fragile X syndrome and glycogen storage disease type IX *Drosophila* genetic disease models demonstrate an essential interaction between *dFMRP* and *dPHKA2* functions, with metabolic and neuromuscular junction defects completely rectified by co-elimination of the encoded proteins.

## INTRODUCTION

Glycogen storage disease (GSD) is the umbrella term for disorders characterized by the inability to break down glycogen, the critical short-term energy store within cells (Stone et al., 2023a; Gümüş and Özen, 2023). One specific disorder is GSD type IX (GSD-IX), also known as X-linked glycogenesis (Kishnani et al., 2019; Hendrickx et al., 1996). A classic patient case (‘Patient 2’) comes from a family with an established GSD-IX history from a phosphorylase kinase regulatory subunit alpha 2 (*PHKA2*) C3614T point mutation (De Boulle et al., 1993). However, Patient 2 presented with symptoms far more severe than those associated with GSD-IX, including lack of communicative abilities, extreme cognitive disabilities (IQ<20), striking anatomical deformities and exceptional macroorchidism (De Boulle et al., 1993). These symptoms are inconsistent with GSD-IX, but characteristic of fragile X syndrome (FXS) (Stone et al., 2023b; Garber et al., 2008), albeit far more severe than typical for a patient with FXS. Indeed, although Patient 2 tested negative for expanded fragile X ribonucleoprotein 1 (*FMR1*) CGG repeats, the usual cause of FXS (Sherman et al., 2005), the patient proved to have a unique *de novo FMR1* missense mutation (I304N; Zang et al., 2009), with isoleucine replacing asparagine in the second KH-type RNA-binding domain of the fragile X ribonucleoprotein 1 (FMRP) translational regulator (Zang et al., 2009; Feng et al., 1997). This discovery sparked the hypothesis that the *FMR1* I304N variation is somehow uniquely detrimental (Schrier et al., 2004; Valverde et al., 2007) and generated prolonged research on *FMR1* I304N disease models (Huebschman et al., 2020; Zang et al., 2009).

FXS is a heritable intellectual disability and autism spectrum disorder (Stone et al., 2023b; Garber et al., 2008). FMRP loss in human patients and mouse models also alters motor function and causes supernumerary immature synapses, with the full nulls exhibiting elevated neurotransmitter release paired with impaired synaptic plasticity (Deng et al., 2013; Sidorov et al., 2013). Autophagy dysfunction in FXS mouse models is associated with synapse transmission and remodeling defects (Zhang et al., 2024; Guo et al., 2023). In contrast, the *Fmr1* I304N mouse model exhibits only partial FMRP loss of function, not the hypothesized gain of function (Zang et al., 2009). Although the *dFmr1* (also known as *Fmr1*) I304N mutation impairs canonical FMRP RNA-binding and polyribosome association translational control, the mutants certainly do not exhibit the greatly heightened Patient 2 defects (Pozdnyakova and Regan, 2005; Feng et al., 1997). In the *Drosophila* FXS model, *dFmr1* null mutants likewise exhibit altered motor function, supernumerary immature synapses and elevated neurotransmission, together with impaired synaptic plasticity (Leahy et al., 2023; Song et al., 2022; Repicky and Broadie, 2009). However, *Drosophila* I304N model studies also demonstrate only partial loss of function (Zang et al., 2009; Wang et al., 2008; Wan et al., 2000) and not the heightened defects characterizing Patient 2. Thus, the catastrophic Patient 2 symptoms are not due to any unique *dFmr1* I304N effects, but are rather presumably attributable to the combined FXS and GSD-IX disease states, with the joint loss of both FMRP and PHKA2 functions within the same patient. We therefore hypothesized an FMRP-PHKA2 interaction based on an unsustainably increased metabolic demand from mismatch between reduced energy store accessibility (GSD-IX) and elevated energy needs (FXS) within the double mutants.

The GSD-IX disease state alone is characterized by hypoglycemia with reduced blood sugar energy supply and hypertriglyceridemia with elevated blood triglycerides (Ross et al., 2020; Tagliaferri et al., 2022; Roscher et al., 2014; Özen, 2007). High-fat diets have been recommended to maximize ketogenic and β-oxidation pathways, as alternatives to glycogen breakdown (Bhattacharya et al., 2016). A *Drosophila* GSD-IX model has not previously been established, but *Drosophila* glycogen is likewise a critical metabolic energy store, with defects in glycogen breakdown linked to triglyceride mobilization and early lethality (Yamada et al., 2018). The *Drosophila* fat body serves multiple roles in the balanced regulation of these energy stores, and has an equivalent biological function to the mammalian liver in the maintenance and utilization of fat and glycogen stores (Lehmann, 2018; Zhang and Xi, 2014). Fat body protein 1 (Fbp1) is a key player in the fat body triglyceride metabolism pathway (Burmester et al., 2001). The Fbp1 receptor binds Larval serum protein 1 and 2 (Lsp1 and Lsp2) to mobilize fat energy stores (Musselman et al., 2013; Burmester et al., 2001), with aberrant glycogen metabolism linked to Fbp1 function through intersecting regulatory metabolic pathways (Gillette et al., 2021). Mitochondrial function is also closely intertwined with fat body metabolic processes (Sriskanthadevan-Pirahas et al., 2022; Baltzer et al., 2009). The impact of dysregulation of fat and glycogen catabolism pathways on mitochondrial function is an important question when testing the metabolic and neurological defects associated with any disease state characterized by alterations in energy store dynamics.

The FXS disease state alone exhibits grossly elevated metabolic demand defects (Dionne et al., 2024; Abbasi et al., 2024; Min et al., 2009). In the mouse FXS model, *FMR1* null mutants have a highly increased glucose metabolism rate (Qin et al., 2002). Metabolomics reveals higher lipid metabolism and mobilization (Leboucher et al., 2019). FMRP loss reduces fat stores in patients with FXS and in animal disease models (Lumaban and Nelson, 2015). In the *Drosophila* FXS model, *dFmr1* null mutants reveal a simultaneous increase in brain insulin signaling, with large metabolic fluctuations driven by either stressful or starvation conditions (Monyak et al., 2017). This disease model also displays linked mitochondrial dysfunction, associated with shifts in metabolic pathways and linked behavioral outputs (Licznerski et al., 2020; Weisz et al., 2018). Specifically, the FXS model has elevated mitochondrial oxidative phosphorylation across the electron transport chain linked to abridged motor skills, and such metabolic abnormalities may explain FXS pathophysiology (Vannelli et al., 2024; Drozd et al., 2018). We hypothesized that FXS model animals are sensitive to metabolic stress due to an elevated need for energy supported by hyperactive metabolic pathways. In addition to fat and glycogen assays, mitochondrial function can be tested via MitoTracker fluorescence levels (e.g. MitoTracker Orange) (Agnello et al., 2008). Fluorescence intensity reveals mitochondrial function (Harwig et al., 2018) in localized subcellular compartments such as synapses [e.g. neuromuscular junction (NMJs)]. Established assays for *Drosophila* NMJ synaptic structure and neurotransmission strength (Eberwein et al., 2023; Leahy et al., 2023, 2024) can be coupled to test the metabolism-dependent neurological consequences in GSD-IX and FXS disease models, alone and in double knockout (DKO) joint model combination.

Given the severity of Patient 2 symptoms (Castrén et al., 2001; Feng et al., 1997), metabolic restriction in GSD models (Ross et al., 2020; Özen, 2007) and high metabolic demand in FXS models (Menzies et al., 2021; Leboucher et al., 2019), we tested here for a negative synergistic interaction between FMRP and PHKA2 functions. We assayed for altered energy store availability in a state of elevated metabolic demand affecting NMJ synaptic properties in the GSD-IX model alone [*dPHKA2* (also known as *CG7766*) null mutant], FXS model alone (*dFmr1* null mutant) and the combined models (DKO). We found that *dPHKA2* and *dFmr1* nulls were fully viable, but DKO caused 100% synthetic lethality, establishing an interaction. As expected, the FXS model displayed a loss of fat stores, whereas the GSD-IX model had elevated fat stores, with the differences canceled out in DKO animals. The *dFmr1* null also had elevated glycogen, which was lost in DKO animals. Interestingly, regulatory Fbp1 was upregulated in both single models but restored to normal in DKO animals. Consistently, NMJ mitochondrial function revealed by MitoTracker fluorescence was higher in all three disease models, suggesting a common, intersecting pathway. NMJ bouton number and synaptic contact area were elevated in *dFmr1* nulls, with increases corrected by *dPHKA2* co-removal in DKO animals. Likewise, neurotransmission strength elevated in *dFmr1* nulls was fully rectified by this *dPHKA2* co-removal. Interestingly, *dPHKA2* overexpression (OE) replicated the *dFmr1* null phenotype, with synaptic function remaining elevated in the double mutant animals. These findings indicate that the joint disease model ameliorates FXS metabolic and neurological defects despite the synthetic lethality interaction.

## RESULTS

### The combined GSD-IX and FXS *Drosophila* disease model is synthetic lethal

**Fig. 1.**
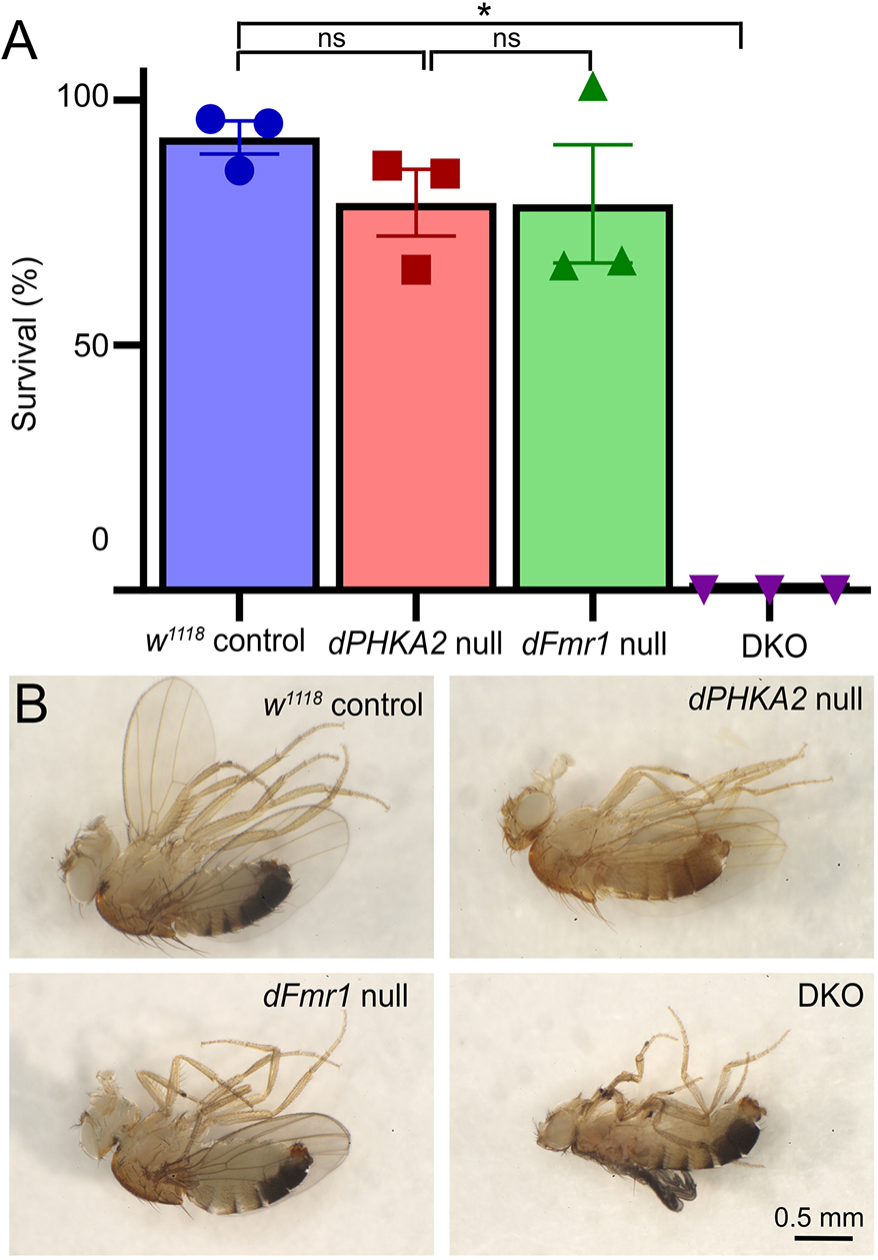
**Glycogen storage disease type IX (GSD-IX) and fragile X syndrome (FXS) disease models combine to cause synthetic lethality.** (A) Adult survival as a percentage of that expected for the genetic background control (*w^1118^*), the GSD-IX disease model (*dPHKA2* null), the FXS disease model (*dFmr1* null) and the combined double knockout (DKO) model. Scatter plots show the data points from three independent trials with the mean±s.e.m. Significance is quantified using Kruskal–Wallis followed by a Dunn's multiple-comparisons test. *P≤0.05; ns, not significant (*P*>0.05). (B) Representative images show recently eclosed juvenile adult males of all four genotypes. Rare DKO escapers eclose at a very low frequency, but always die as young juveniles. The flies are shown on their sides, with anterior to the left, posterior to the right, and legs projecting upwards. Scale bar: 0.5 mm.

We hypothesized that GSD-IX and FXS disease models would exhibit a negative synergistic interaction based on heightened and unmet metabolic demands. We therefore predicted that combining *dPHKA2* and *dFmr1* null mutations would lead to more severe phenotypes than each single disease model alone. To test this hypothesis, we compared the genetic background control (*w^1118^*), GSD-IX model [*dPHKA2^CRI/^*^Y^ null; Bloomington *Drosophila* Stock Center (BDSC) 79254], FXS model (*dFmr1^50M/3^* null; Zhang et al., 2001) and the joint combined disease model (*dPHKA2^CRI/^*^Y^; *dFmr1^50M/3^*), referred to as the DKO. This is the first reported characterization of the *Drosophila* GSD-IX disease model, but the *Drosophila* FXS disease model is established and well characterized as adult viable and morphologically normal (Song et al., 2022; Vita et al., 2021; Sears and Broadie, 2020; Zhang et al., 2001). We first assayed the overall genotype viability and morphological development. All four genotypes were reared side by side under standard temperature (25°C) and light (12-h light/dark) conditions. The percentage survival at adult eclosion was scored relative to the expected number for each genotype (Kumari et al., 2023). We also tested survival with a complex carbohydrate diet supplement used to treat patients with GSD (Ross et al., 2020), comparing genotypes raised on simple dextrose or complex starch food. DKO animals could successfully eclose, when reared at low density with exceptional care. Adult morphology at this end-point terminal stage (<12 h post-eclosion) was imaged with a dissection microscope and compared among all four genotypes. Quantified survival data and representative adult images are shown in Fig. 1.

Compared to controls, single *dPHKA2* and *dFmr1* nulls did not exhibit significant survival impairments (Fig. 1A). There were no significant adult survival differences for either *dPHKA2* (79.08±6.83%, *n*=3 trials; *P*=0.91) or *dFmr1* (78.85±12.04%, *n*=3; *P*>0.9999) null mutants compared to *w^1118^* control animals (92.41*±*3.35%*; n=*3; Fig. 1A). In contrast, DKO animals showed complete lethality (0% survival rate, *n*=3; *P*=0.026; Fig. 1A). The *dPHKA2* nulls reared on a complex carbohydrate diet developed faster and exhibited greater survival than mutants on simple dextrose food (55.65%). However, DKO animals continued to show complete lethality on both diets. Newly eclosed controls had well developed abdomens, thoracic legs and wings, and an appropriate adult anatomy characteristic of completed metamorphosis (Fig. 1B, top left). Single *dPHKA2* and *dFmr1* nulls did not have detectable anatomical impairments, so both GSD-IX and FXS disease models were indistinguishable from the *w^1118^* background control (Fig. 1B). In contrast, the combined disease model exhibited striking anatomical defects. Very rare DKO escapers could be found by examining large numbers of vials of recently eclosed flies. These DKO animals had deformed legs with dark coloration, particularly around the joints (Fig. 1B, bottom right). DKO animals also failed to inflate their wings, which were deformed and smaller than those of controls (Fig. 1B, bottom right). DKO movement showed severe motor deficits in coordination and general locomotion, with defects presumably preventing adult eclosion in almost all cases and rapid post-eclosion lethality. Thus, the GSD-IX and FXS disease models negatively interact to cause complete synthetic lethality and anatomical defects. We therefore next tested whether this interaction occurs through heightened and unmet metabolic demands.

### The combined GSD-IX and FXS disease model balances fat and glycogen store defects

**Fig. 2.**
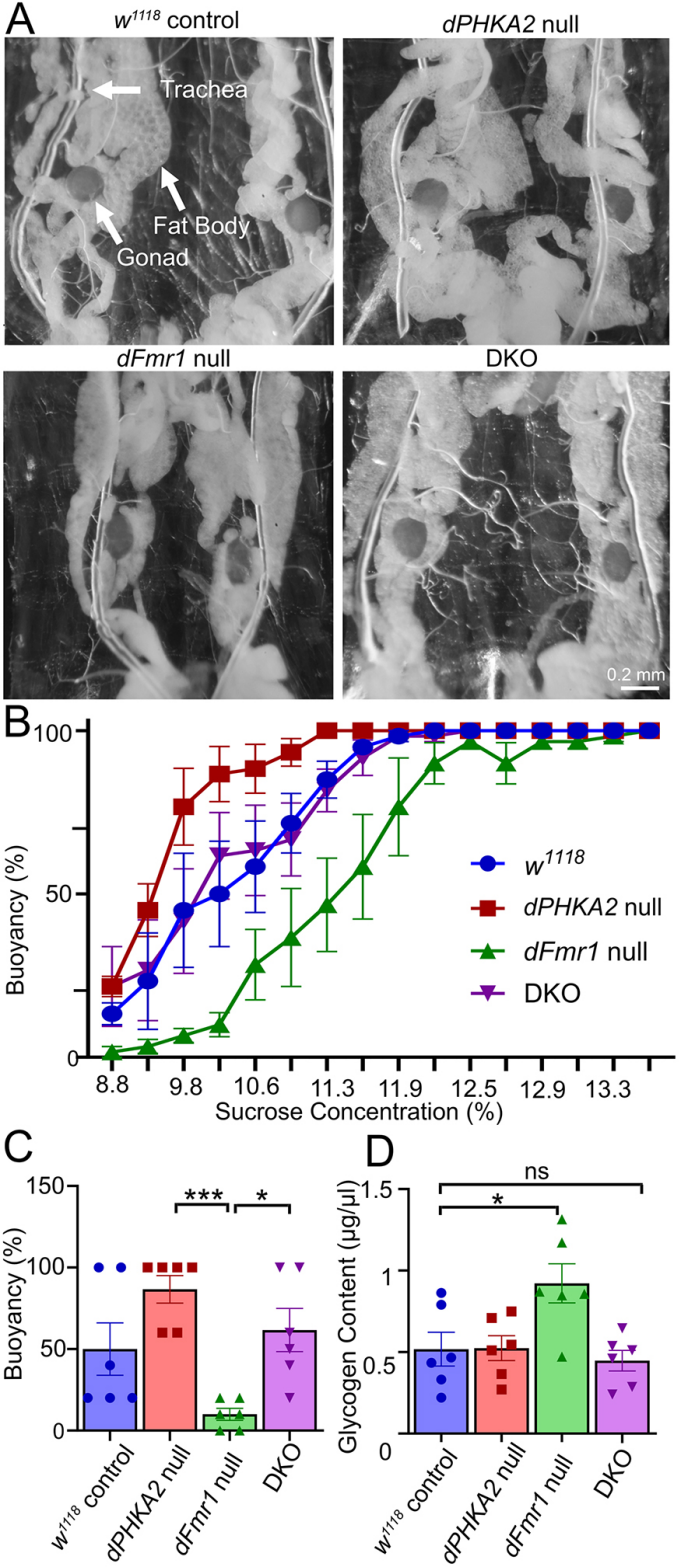
**Combined disease model balances glycogen and fat metabolic stores.** (A) Representative wandering third-instar dissections for the background control (*w^1118^*), *dPHKA2* null (*PHKA2^Cri^*), *dFmr1* null (*dFmr1^50M/3^*) and the DKO to show fat accumulation. Arrows indicate fat body, trachea and gonads as labeled. Scale bar: 0.2 mm. (B) Buoyancy-based fat quantification for all four genotypes with the percentage buoyancy relative to the sucrose concentration. (C) Percentage buoyancy at the midpoint of the sucrose concentration curve (10.2%) compared using Kruskal–Wallis followed by a Dunn's multiple-comparisons test. (D) Glycogen quantification compared using one-way ANOVA followed by a Dunnett's multiple-comparisons test. Histogram scatter plots show all data points with mean±s.e.m. ***P≤0.001, *P≤0.05; ns, not significant (*P*>0.05).

GSD is associated with increased risk of hypoglycemia and hypertriglyceridemia (Ross et al., 2020; Bhattacharya et al., 2016; Roscher et al., 2014). The mouse FXS model has elevated glucose and lipid metabolism (Qin et al., 2002), with a heightened metabolic flux (Leboucher et al., 2019). However, fat and glycogen stores in the two larval *Drosophila* disease models have not been characterized. Given that *dPHKA2* and *dFmr1* double nulls showed pharate adult lethality (Fig. 1), all subsequent studies were done in wandering third instars to late-stage pupae. To test for potential metabolic links between *dPHKA2* and *dFmr1*, fat and glycogen stores were assessed in *w^1118^* background control, GSD-IX model alone, FXS model alone and the combined DKO disease model. Fat stores were imaged directly and also quantified with a buoyancy assay in the wandering third instars (Hazegh and Reis, 2016). The percentage buoyancy across a range of sucrose concentrations was tested, with larvae containing greater fat stores more buoyant at lower sucrose concentrations. For statistical interpretations, buoyancy values at the midpoint of the concentration range were compared between all four genotypes. Glycogen stores were next tested with a colorimetric assay in the wandering third instars. Replicates of background controls were subtracted from raw sample data for each genotype before calculating glycogen content relative to a standard curve specific for each independent trial. Representative images and quantifications are shown in Fig. 2.

Dissected wandering third instars with exposed fat bodies are shown in Fig. 2A. The *w^1118^* background controls have moderate levels of fat, compared to those of *dPHKA2* nulls, with larger and denser fat deposits (Fig. 2A, top row). In contrast, *dFmr1* nulls had much less fat than controls. Interestingly, DKO animals were visually indistinguishable from the background control, suggesting no difference in energy stores (Fig. 2A, bottom row). Next, a buoyancy-based assay was used to quantify fat store levels in all four genotypes (Fig. 2B). Compared to *w^1118^* background controls (blue, Fig. 2B), the *dPHKA2* nulls had higher buoyancy across the sucrose concentration gradient (red, Fig. 2B), the *dFmr1* nulls had lower buoyancy across the sucrose concentration gradient (green, Fig. 2B), and the buoyancy levels of DKO animals were completely indistinguishable from those of the controls (purple, Fig. 2B). Quantifying fat buoyancy at the control sucrose concentration curve midpoint (10.2%) showed that *dPHKA2* nulls had strikingly higher fat stores (86.67±8.43, *n*=6; *P*=0.0008), compared to *dFmr1* nulls, which had greatly reduced fat stores (10.0±3.65, *n*=6; *P*=0.024) (Fig. 2C). The DKO animals (61.67±13.27, *n*=6) were comparable to controls (50.00±16.12, *n*=6; *P*>0.999) (Fig. 2C). For the glycogen colorimetric assay, compared to controls (0.52±0.104 μg/μl, *n*=6), *dFmr1* nulls displayed significantly greater glycogen stores (0.92±0.12 μg/μl, *n*=6; *P*=0.017) (Fig. 2D). Surprisingly, *dPHKA2* nulls exhibited no significant change compared to control (0.52±0.076 μg/μl, *n*=6; *P*=0.999). However, the DKO animals had completely restored normal glycogen levels (0.45±0.064 μg/μl, *n*=6; *P*=0.91) and were comparable to controls (Fig. 2D). These findings suggest a mismatch between energy stores in GSD-IX versus FXS disease models, which is rebalanced when the models are combined.

**Fig. 3.**
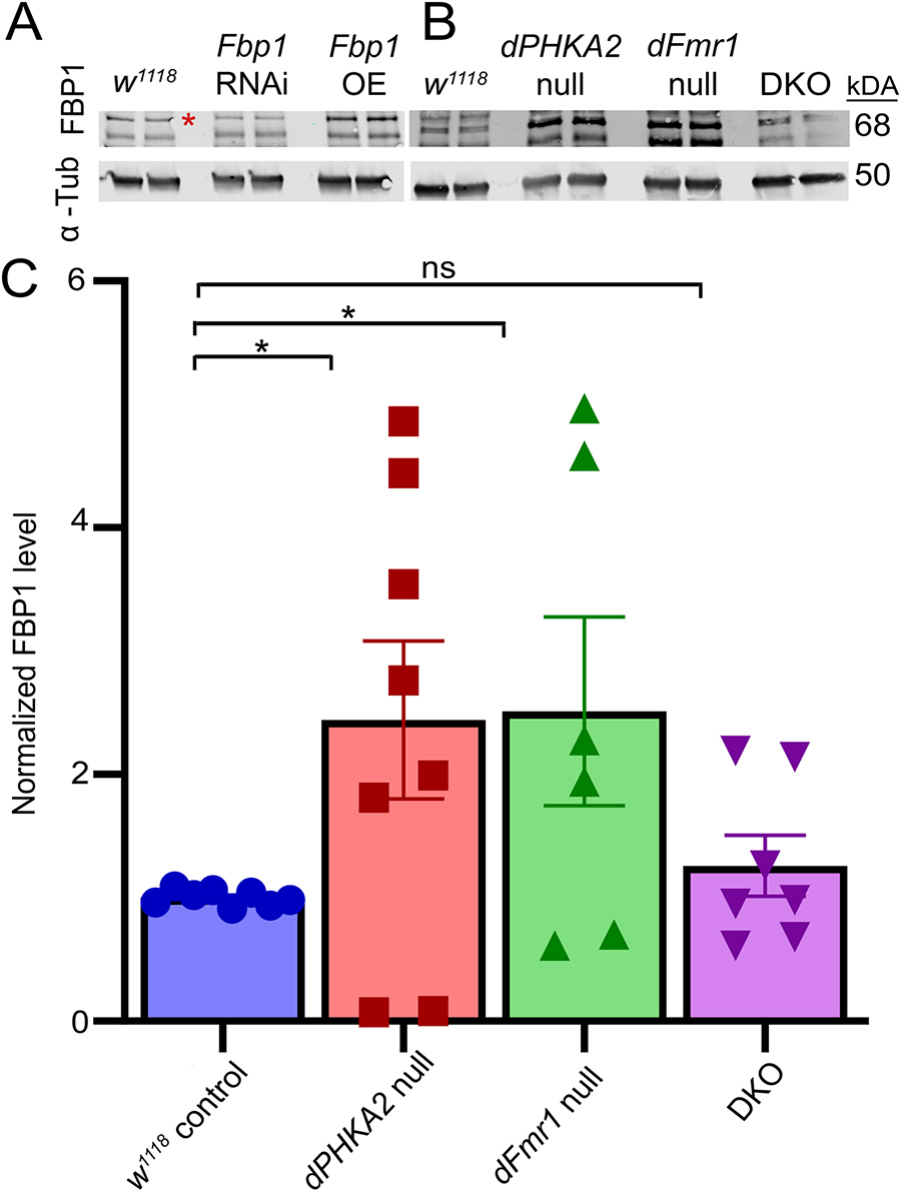
**Fat body protein 1 (Fbp1) receptor levels elevated in single disease models are rectified in DKO.** (A) Representative western blot of anti-Fbp1 and α-Tubulin (α-Tub) in the driver control (*w^1118^*×UH1-Gal4), *Fbp1* RNA interference (RNAi) and *Fbp1* overexpression (OE) driven ubiquitously by UH1-Gal4 in pharate adults. Multiple immunoreactive bands are present, with the red asterisk marking the Fbp1 band used for quantification in C. Two independent trial lanes are shown for each of the genotypes. (B) Representative western blot of Fbp1 and α-Tub for the genetic background control (*w^1118^*), *dPHKA2* null (*PHKA2^Cri^*), *dFmr1* null (*dFmr1^50M/3^*) and DKO pharate adults. Two independent trial lanes are shown for each of the four genotypes. (C) Fbp1 levels are normalized to α-Tub and the control, and statistically compared using unpaired two-tailed *t*-tests after outliers removed. The scatter plot shows all the data points with mean±s.e.m. *P≤0.05; ns, not significant (*P*>0.05).

We next tested regulatory mechanisms for metabolic store mobilization in all four genotypes. Fbp1 acts as a receptor for Lsp1 and Lsp2 ligands to mobilize fat energy stores in close coordination with glycogen regulation (Burmester et al., 2001). Fbp1 western blots showed multiple bands around the predicted molecular mass (68 kDa; Fig. 3A), with several different known isoforms and post-translational modifications (Burmester et al., 2001). Antibody specificity was shown with *Fbp1* RNA interference (RNAi) knockdown and *Fbp1* OE driving elevated protein levels (Fig. 3A). Compared to the levels in the *w^1118^* background control, Fbp1 levels were clearly elevated in both *dPHKA2* and *dFmr1* null mutants (Fig. 3B). In contrast, Fbp1 levels in the joint DKO disease model were comparable to those in controls (Fig. 3B). Quantified levels were normalized to an α-Tubulin loading control for each genotype. Compared to the levels in the *w^1118^* controls (1.00±0.022, *n*=8), the Fbp1 levels were significantly elevated in both *dPHKA2* (2.44±0.64, *n*=8, *P*=0.041) and *dFmr1* (2.51±0.77, *n*=6; *P*=0.039) null mutants (Fig. 3C). This elevation was completely prevented in the combined DKO disease model, with Fbp1 levels comparable to those in controls (1.26±0.25, *n*=7) and no significant difference remaining (*P*=0.28; Fig. 3C). These findings suggest that both single disease models require increased fat breakdown to meet metabolic demands, whereas the DKO disease model rebalances the same pathway to accommodate metabolic demand. We next turned to testing phenotypic outputs for this altered metabolic processing in both single and double disease models, assaying consequences at the well-characterized NMJ.

### GSD-IX and FXS disease models show elevated NMJ synaptic mitochondrial function

**Fig. 4.**
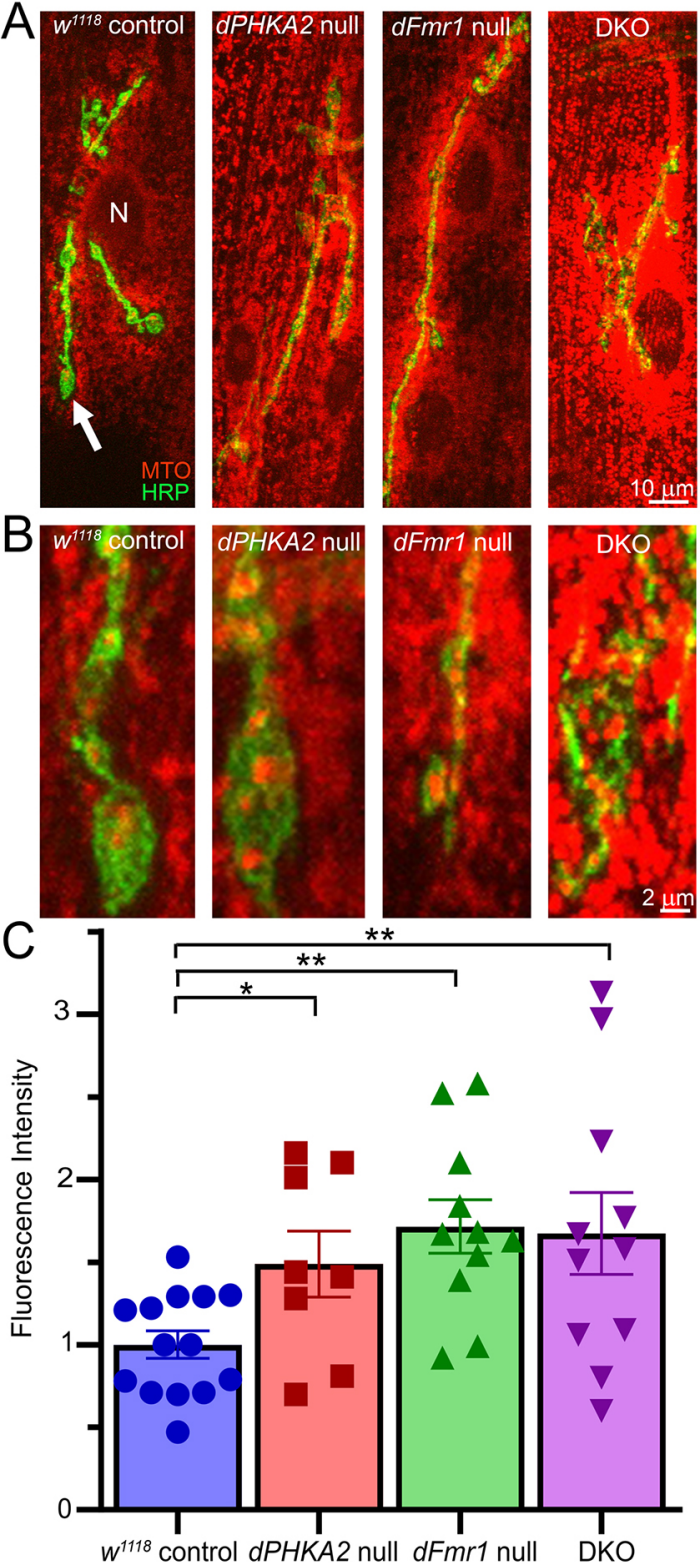
**Neuromuscular junction (NMJ) synaptic mitochondrial function is elevated in all the disease models.** (A) Representative confocal images of wandering third-instar NMJ synaptic terminals co-labeled with MitoTracker Orange (MTO) and presynaptic membrane marker anti-horseradish peroxidase (HRP; green) to show the NMJ (arrow). Muscle 4 NMJs shown from left to right: the genetic background control (*w^1118^*, *n*=14), *dPHKA2* null (*PHKA2^Cri^*, *n*=8), *dFmr1* null (*dFmr1^50M/3^*, *n*=11) and DKO (*n*=11) condition. ‘N’ indicates muscle nucleus; arrow indicates NMJ bouton. Scale bar: 10 µm. (B) Higher-magnification images of NMJ boutons in the above four genotypes. Scale bar: 2 µm. (C) MitoTracker fluorescence intensities within the HRP-delineated NMJ compared with a one-way ANOVA followed by Holm-Šidȧk's multiple comparisons test after outliers removed. The scatter plot shows all data points with mean±s.e.m. **P≤0.01, *P≤0.05; ns, not significant (*P*>0.05).

Because metabolic pathways are disrupted in both GSD-IX and FXS disease models, we next sought to assess metabolically demanding motor function. The *Drosophila* FXS model exhibits altered movement (Kashima et al., 2017; Zhang et al., 2005), with the controlling NMJ particularly well characterized at many levels (Leahy et al., 2024; Menon et al., 2013; Chou et al., 2020), including mitochondrial activity (Mallik and Frank, 2022). The *Drosophila* FXS model shows mitochondrial functional changes (Pagano et al., 2024; Vannelli et al., 2024; Licznerski et al., 2020; Weisz et al., 2018; Napoli et al., 2016), but the GSD-IX model had not yet been tested. We labeled *Drosophila* NMJs with anti-horseradish peroxidase (HRP) (Bhimreddy et al., 2021; Eberwein et al., 2023) and used the fixable MitoTracker Orange sensor to assay NMJ mitochondrial function (Harwig et al., 2018; Agnello et al., 2008). MitoTracker fluorescence clearly revealed mitochondrial function in and surrounding NMJ terminals (Fig. 4A). In the background control, red fluorescent puncta marking mitochondria in NMJ boutons and in surrounding muscle showed the normal level of mitochondrial function (Fig. 4B). The NMJs of all three disease models showed elevated MitoTracker fluorescence compared to controls (Fig. 4A,B). This heightened function was both within the NMJ and in surrounding postsynaptic muscle. Quantified MitoTracker fluorescence compared to that of the *w^1118^* control (normalized 1.00±0.16, *n*=14) showed a less significant increase in *dPHKA2* nulls (1.49±0.20, *n*=8; *P*=0.048) and greater increase in *dFmr1* nulls (1.72±0.16, *n*=11; *P*=0.0069) (Fig. 4C). Interestingly, the combined DKO animals continued to show significantly elevated MitoTracker fluorescence at the NMJ (1.67±0.25, *n*=11; *P*=0.0077) compared to the controls (Fig. 4C). These findings suggest that both GSD-IX and FXS disease models elevate NMJ mitochondrial function, with persistent increased activity in the DKO model. Tests for changes in local reactive oxygen species (ROS) at the NMJ or global ATP levels failed to reveal any significant differences between any of the four genotypes (Fig. S1). We next turned to testing NMJ phenotypes in single and double disease models through assaying synaptic architecture and function.

### FXS disease model supernumerary synaptic boutons are corrected by *dPHKA2* co-removal

**Fig. 5.**
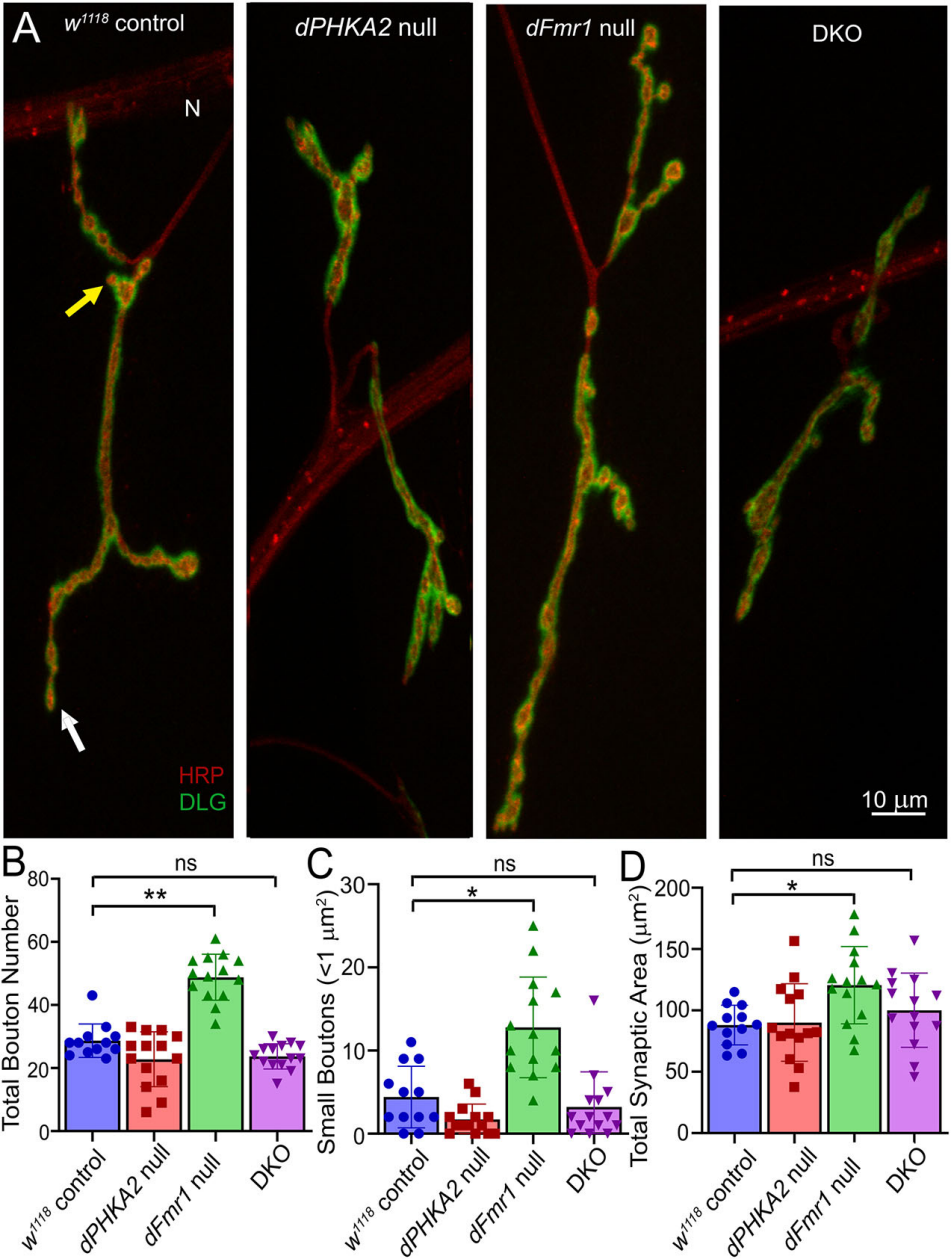
**FXS synaptic overgrowth is corrected by *dPHKA2* co-removal*.*** (A) Representative confocal images of wandering third-instar NMJs co-labeled with presynaptic anti-HRP (red) and postsynaptic anti-Discs large (DLG; green) to show the synaptic architecture. Scale bar: 10 µm. Muscle 4 NMJs shown for genetic background control (*w^1118^*, *n*=12), *dPHKA2* null (*PHKA2^Cri^*, *n*=14), *dFmr1* null (*dFmr1^50M/3^*, *n*=14) and DKO (*n*=14). Scale bar: 10 µm. ‘N’ indicates the motor nerve; the white arrow indicates a mature synaptic bouton, and the yellow arrow indicates a mini-bouton. (B-D) Quantifications of total bouton number (B), mini-bouton number (C), and total synaptic area (D), analyzed using Kruskal–Wallis with Dunn's multiple-comparisons test. Scatter plots show all data points with mean±s.e.m. **P≤0.01, *P≤0.05; ns, not significant (*P*>0.05).

Alterations in mitochondrial function are linked to aberrant synapse architecture, including changes in synaptic bouton number and contact area (Mallik and Frank, 2022; Graham et al., 2017). In patients with FXS and mouse models, FMRP is a negative regulator of synaptogenesis (Clifton et al., 2020; Bassell and Warren, 2008). In the *Drosophila* FXS model, *dFmr1* nulls likewise show increased NMJ branching and bouton numbers (Siller and Broadie, 2011; Pan et al., 2004; Zhang et al., 2001). In addition to mature NMJ boutons, *dFmr1* nulls also produce supernumerary small, mini-boutons thought to be immature contacts in the process of forming (Gatto and Broadie, 2008). In contrast, *dPHKA2* roles in synaptic architecture have not previously been investigated. Because Patient 2 showed neurological impairments far more severe than the FXS disease condition alone (Castrén et al., 2001; Feng et al., 1997; De Boulle et al., 1993), we hypothesized that an FMRP-PHKA2 negative interaction influences synaptic architecture. We therefore tested wandering third instars by co-labeling presynaptic boutons with anti-HRP and muscle postsynaptic domains with anti-Discs large (DLG; also known as Dlg1) to specifically identify the type 1b synaptic boutons (Leahy et al., 2023, 2024). We quantified total and mini (<1 µm) boutons, as well as total synaptic contact area. The *w^1118^* genetic background control was compared to GSD-IX and FXS single null mutant models, as well as the combined DKO disease model. Representative images and quantifications are shown in Fig. 5.

The *w^1118^* controls showed reproducible synaptic architecture, with regular branches, a well-spaced array of mature synaptic boutons and the rare mini-boutons (Fig. 5A). The *dPHKA2* nulls were comparable to controls and did not show any striking changes (Fig. 5A). In contrast, the *dFmr1* nulls exhibited obvious changes across multiple synaptic parameters, with obvious branching overgrowth and supernumerary boutons (Fig. 5A). Interestingly, DKO animals showed none of these features, but rather displayed a synaptic architecture like that of controls (Fig. 5A). Quantification of the total NMJ bouton number compared to that of *w^1118^* controls (28.67±1.53, *n*=12) revealed no change in the *dPHKA2* nulls (22.79±2.32, *n*=14; *P*>0.999), a significant elevation in the *dFmr1* nulls (48.79±1.95, *n*=14; *P*=0.006), and a complete rectification back to control levels in DKO animals (23.71±1.07, *n*=14; *P*=0.54) (Fig. 5B). Imaging small mini-boutons compared to those in *w^1118^* controls (4.42±1.069, *n*=12) similarly revealed no significant change in *dPHKA2* nulls (1.71±0.50, *n*=14; *P*=0.49), significantly greater numbers in *dFmr1* nulls (12.79±1.62, *n*=14; *P*=0.022) and correction to the control levels in DKO animals (3.21±1.13, *n*=14; *P*>0.999) (Fig. 5C). Quantification of synaptic contact area compared to that in *w^1118^* controls (88.06±4.66 µm^2^, *n*=12) likewise showed no change in *dPHKA2* nulls (90.12±8.44 µm^2^, *n*=14; *P*>0.999), a significant increase in *dFmr1* nulls (120.60±8.42 µm^2^, *n*=14; *P*=0.043) and a restoration to control levels in DKO animals (100.20±8.08 µm^2^, *n*=14; *P*>0.999) (Fig. 5D). Importantly, control and DKO animals did not differ significantly in any synaptic measurement (*P*>0.999; Fig. 5B-D). The opposing *dPHKA2* OE did not significantly alter the elevated synaptic boutons of the FXS model (Fig. S2A); with *dPHKA2* OE, *dFmr1* null total bouton number (49.36±3.85, *n*=14) remained significantly elevated compared to that in controls (29.57±2.00, *n*=14; *P*=0.0004; Fig. S2B). In contrast, the heightened number of mini-boutons in the FXS model was restored towards normal in the double mutant condition (Fig. S2C). We next tested functional synaptic transmission in single and double mutant disease models.

### FXS disease model elevated neurotransmission is rectified by *dPHKA2* co-removal

**Fig. 6.**
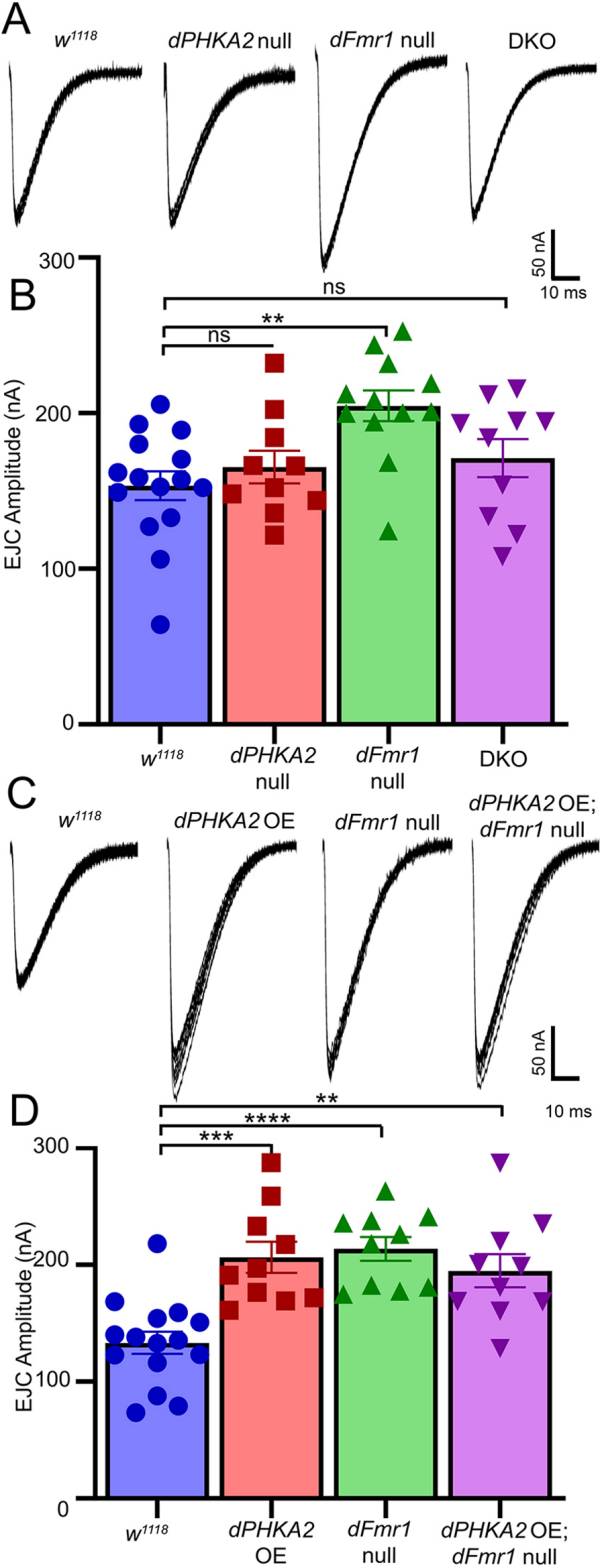
**FXS-strengthened neurotransmission is rectified by *dPHKA2* co-removal.** Representative wandering third-instar NMJ nerve stimulation-evoked excitatory junction current (EJC) traces shown as ten superimposed responses (1.0 mM Ca^2+^) from left to right for genetic background control (*w^1118^*, *n*=15), *dPHKA2* null mutant (*PHKA2^Cri^*, *n*=10), *dFmr1* null mutant (*dFmr1^50M/3^*, *n*=12) and the DKO (*n*=10) condition. (B) Quantified EJC amplitudes for all four genotypes. (C) Representative EJC traces for control (*w^1118^*), *dPHKA2* OE (*PHKA2^Cri/EP^*), *dFmr1* null (*dFmr1^50M/3^*), and *dFmr1* null with *dPHKA2* OE (*PHKA2^Cri/EP^*; *dFmr1^50M/3^*). (D) Quantified EJC amplitudes for all four genotypes, analyzed using one-way ANOVA followed by Tukey's multiple comparisons test. Scatter plots show all data points with mean±s.e.m. ****P≤0.0001, ***P≤0.001, **P≤0.01; ns, not significant (*P*>0.05).

**Fig. 7.**
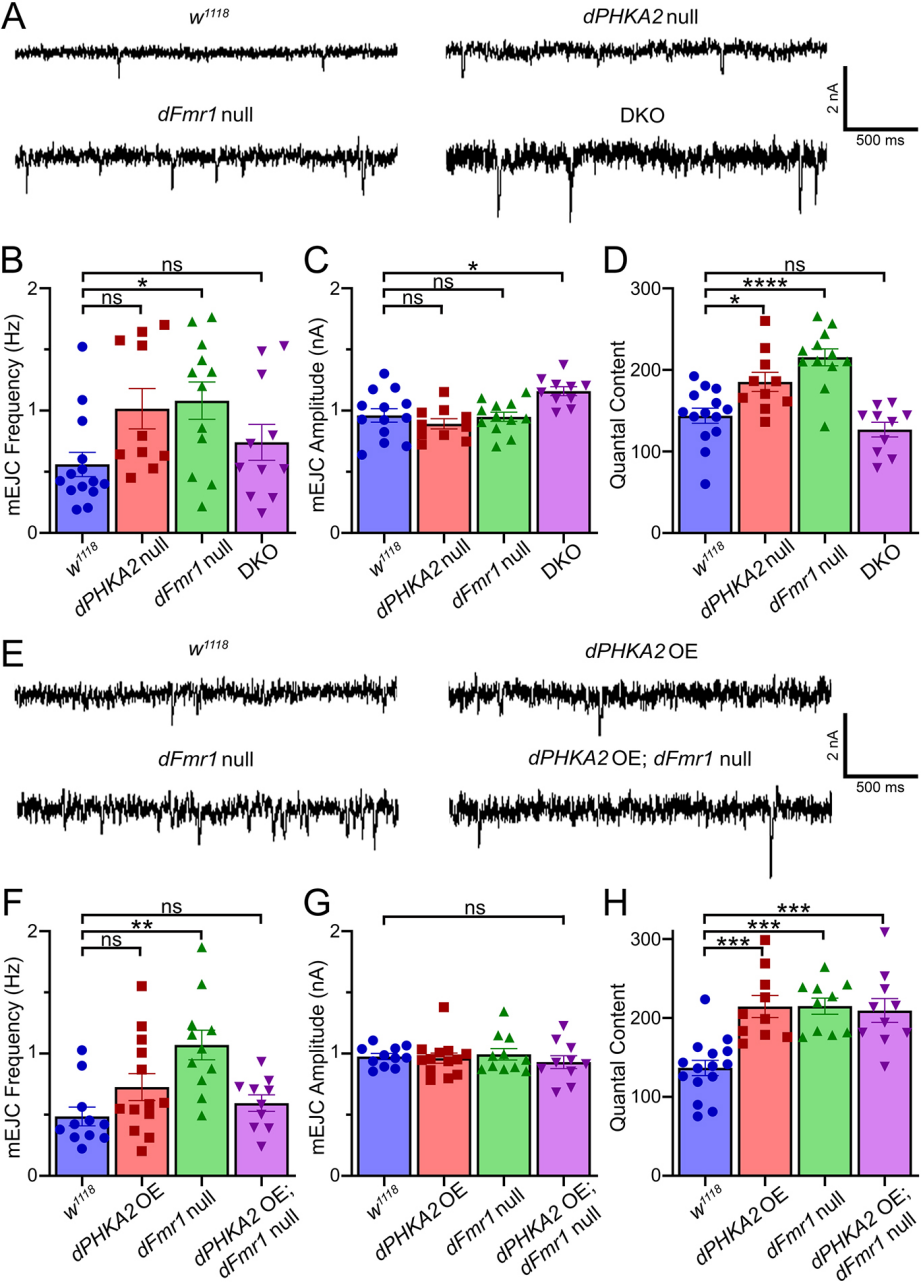
**FXS-elevated quantal fusion and content are rectified by *dPHKA2* co-removal.** (A) Representative wandering third-instar NMJ spontaneous miniature excitatory junction current (mEJC) traces (1.0 mM Ca^2+^) from left to right: background control (*w^1118^*, *n*=14), *dPHKA2* null mutant (*PHKA2^Cri^*, *n*=10), *dFmr1* null mutant (*dFmr1^50M/3^*, *n*=12) and DKO (*n*=10). (B-D) Quantified mEJC frequency (B), amplitude (C) and quantal content (D). (E) Representative mEJC traces for control (*w^1118^*, *n*=11), *dPHKA2* OE (*PHKA2^Cri/EP^*, *n*=14), *dFmr1* null (*dFmr1^50M/3^*, *n*=11), and *dFmr1* null with *dPHKA2* OE (*PHKA2^Cri/EP^*; *dFmr1^50M/3^*, *n*=10). (F-H) Quantified mEJC frequency (F), amplitude (G) and quantal content (H). Statistical analysis was performed using one-way ANOVA followed by Tukey's multiple comparisons test. Scatter plots show all data points with mean±s.e.m. ****P≤0.0001, ***P≤0.001, **P≤0.01, *P≤0.05; ns, not significant (*P*>0.05).

Synaptic structure and function are independently regulated; connectivity changes cannot predict changes in neurotransmission strength and vice versa. Therefore, the above synaptic architecture interactions did not alter our hypothesis that combining *dPHKA2* and *dFmr1* nulls would lead to more severe neurotransmission phenotypes than either single disease model alone. The *Drosophila* FXS model exhibits elevated NMJ synaptic function (Leahy et al., 2023; Friedman et al., 2013; Siller and Broadie, 2011), but the GSD-IX model has not been tested, and effects in the combined DKO disease model were unknown. Using two-electrode voltage-clamp (TEVC) electrophysiology, we tested motor nerve stimulation evoked excitatory junction current (EJC) amplitudes, as a direct, linear measure of NMJ transmission strength (Eberwein et al., 2023; Bhimreddy et al., 2021). We used suction electrode nerve stimulation (0.5 ms suprathreshold stimuli, 0.2 Hz) onto voltage-clamped muscle 6 (−60 mV), with each single data point reflecting the average of ten sequential responses (Leahy et al., 2023, 2024). In parallel, we recorded spontaneous miniature EJC (mEJC) event frequency and amplitude. Once again, we compared *w^1118^* background control, GSD-IX and FXS models, and the combined DKO disease model. *dPHKA2* OE (Tseng and Hariharan, 2002) was also assayed alone and in combination with the FXS model. Representative EJC/mEJC traces and quantified results across all genotypes are shown in Figs 6 and 7.

The *w^1118^* controls showed high-fidelity NMJ neurotransmission with consistent EJC amplitudes upon repeated motor nerve stimulation (Fig. 6A, left). The *dPHKA2* nulls had indistinguishable synaptic function from that of controls, but *dFmr1* nulls displayed elevated EJC amplitudes (Fig. 6A, middle). Most interestingly, DKO animals restored neurotransmission strength to control level (Fig. 6A). Control EJC amplitudes (153.40±9.26 nA, *n*=15) were not significantly different from those of *dPHKA2* nulls (165.30±10.48 nA, *n*=10; *P*=0.84), but *dFmr1* nulls had significantly elevated EJC amplitudes (204.70±9.84 nA, *n*=12, *P*=0.003) (Fig. 6B). EJC amplitudes in DKO animals (171.20±12.26 nA, *n*=10; *P*=0.61) were returned to control levels based a one-way ANOVA and Tukey's multiple comparisons (*P*=0.0052, Fig. 6B). In the opposing *dPHKA2* OE condition, synaptic transmission strength was clearly elevated to phenocopy the *dFmr1* nulls compared to the *w^1118^* controls (Fig. 6C). Additionally, *dPHKA2* OE in the *dFmr1* null background (*PHKA2A^Cri/EP^*; *dFmr1^50M/3^*) also displayed this elevated synaptic function (Fig. 6C). When quantified compared to EJC amplitudes in controls (133.30±9.52 nA, *n*=15), *dPHKA2* OE alone (206.40±13.34 nA, *n*=10, *P*=0.0002), the *dFmr1* null (213.70±10.19 nA, *n*=10, *P*<0.0001) and *dPHKA2* OE in the *dFmr1* null background (194.90±14.14 nA, *n*=10; *P*=0.0021) all displayed increased EJC amplitudes (Fig. 6D). All three genotypes showed significantly elevated EJC amplitudes compared to those of controls when cross-compared via one-way ANOVA and Tukey's multiple comparison tests (*P*<0.0001; Fig. 6D). mEJC recordings showed very similar results, with the elevated mEJC frequency in the FXS model restored by simultaneous manipulation of *dPHKA2* (Fig. 7A,B,E,F). The mEJC amplitudes changed little across all of the compared genotypes (Fig. 7C,G), with the elevated quantal content of the FXS model corrected by *dPHKA2* co-removal, but not affected by *dPHKA2* OE (Fig. 7D,H). Taken together, these findings indicate a *dFmr1*-*dPHKA2* interaction pathway in the NMJ functional regulation of presynaptic neurotransmitter signaling.

## DISCUSSION

Based on the classic Patient 2 comorbidity of *PHKA2* and *FMR1* double condition (De Boulle et al., 1993), and the lack of any evidence for *Fmr1* I304N gain of function (Zang et al., 2009; Wang et al., 2008; Wan et al., 2000), we hypothesized a negative synergistic *PHKA2-FMR1* interaction based on an unsustainably increased metabolic demand from impaired energy store accessibility (*PHKA2*) coupled to elevated energy needs (*FMR1*). We tested this hypothesis in single and joint GSD-IX and FXS *Drosophila* disease models (Song et al., 2022; Zhang et al., 2001). Although the synthetic lethality and anatomical defects in the combined DKO disease model strongly support our hypothesis (Fig. 1), highly interactive metabolic and neurological defects paradoxically run entirely counter to this hypothesis. We find that DKO animals correct the imbalanced fat stores of *dPHKA2* and *dFmr1* mutants, and elevated glycogen in the *dFmr1* null mutants (Fig. 2). Likewise, the greatly elevated Fbp1 metabolic regulator (Musselman et al., 2013; Burmester et al., 2001) in both single mutants is restored to normal in the DKO animals (Fig. 3). At the NMJ, we find elevated mitochondrial function in both GSD-IX and FXS disease models, which uniquely remains increased in the combined DKO model (Fig. 4), albeit no higher. Finally, we discover that synaptic phenotypes in *dFmr1* null mutants – both the supernumerary synaptic boutons (Fig. 5) and elevated neurotransmission strength – are strongly rectified by co-removal of *dPHKA2* (Figs 6 and 7). These findings demonstrate that GSD-IX and FXS disease models do indeed very strongly interact, albeit not by the means we had hypothesized.

Neither *dPHKA2* nor *dFmr1* nulls show any significant lethality in the solo disease models, but DKO in the combined disease model causes a complete synthetic lethality (Fig. 1A). DKO animals mostly arrest as pharate adults prior to eclosion, a particularly challenging metabolic and behavioral transition point (Chatterjee and Perrimon, 2021). Similarly, Patient 2 with both GSD-IX and FXS possessed symptoms far more severe than those associated with either disease alone (Castrén et al., 2001; Feng et al., 1997; De Boulle et al., 1993). For a very long time, this heightened severity was wholly attributed to the unique *FMR1* I304N point mutation (Huebschman et al., 2020; Zang et al., 2009; Valverde et al., 2007; Schrier et al., 2004); however, work in mouse and *Drosophila* I304N models has failed to replicate the severity of symptoms in the patient (Zang et al., 2009; Wang et al., 2008; Wan et al., 2000). *Drosophila* metamorphosis is known to be energetically costly as the metabolic rate rapidly elevates prior to eclosion, with >80% of the energy derived from breakdown of fat stores (Merkey et al., 2011). Thus, the DKO disease model synthetic lethality is consistent with our metabolic hypothesis. Single *dPHKA2* and *dFmr1* nulls are morphologically normal in both individual disease models, but DKO in the joint disease model causes striking anatomical defects (Fig. 1B). This is consistent with the similarly heightened, extreme anatomical symptoms characterizing Patient 2 (De Boulle et al., 1993). One possible explanation for these defects in the DKO combined disease model could be the mismatch of energy stores with metabolic needs, which would be exacerbated during the challenging metamorphosis period (Chatterjee and Perrimon, 2021; Merkey et al., 2011), leading to improper morphological development and primarily pre-adult lethality.

The GSD-IX model exhibits elevated fat stores, whereas the FXS model shows fat depletion (Fig. 2A-C). These results are consistent with the hypothesis of the metabolic basis of disease interaction and Patient 2 symptom severity (Castrén et al., 2001; Feng et al., 1997; De Boulle et al., 1993). DKO fat stores, however, are comparable to those of controls, undermining the impaired lipid breakdown mechanism (Mendoza-Grimau et al., 2024). Although *dPHKA2* nulls surprisingly show no effect on glycogen stores, *dFmr1* null larvae have elevated glycogen, which is likewise completely corrected in the DKO (Fig. 2D). Importantly, *Drosophila* FXS model adults have lower triglyceride levels and glycogen stores than those of controls, indicating a metabolically distinct state (Weisz et al., 2018; Çaku et al., 2017; Koyama et al., 2020; Rehman and Varghese, 2021). GSD-IX can affect glycogen stores in both liver and muscle (Kishnani et al., 2019; Hendrickx et al., 1996), which prompts the question why we failed to detect a change in our GSD-IX disease model. More work is required to test *dPHKA2* function in relation to glycogen storage regulation. Altered short- and long-term energy stores are inconsistently correlated with both human disease states and previous animal modeling. For example, the *Drosophila* FXS model shows elevated insulin signaling, which should reduce glycogen phosphorylase activity (Post et al., 2018; Bu and Zhang, 2017), but DKO animals display normal glycogen stores. Thus, although GSD-IX and FXS models display distinctly different metabolic states, in fat and glycogen stores, a mismatch is evident, countering our hypothesis. Moreover, we find that the DKO model ameliorates neurological defects associated with the FXS model, characterized by heightened dependence on short-term energy stores via elevated glucose metabolism and tolerance (Leboucher et al., 2019; Qin et al., 2002). A shift to long-term energy stores in the DKO animals may cause the observed synthetic lethality.

Given the altered metabolic stores in both disease models, we sought to assess disturbances in metabolic regulatory pathways (Dionne et al., 2024). The *dFmr1* nulls have elevated Fbp1 levels, and, surprisingly, *dPHKA2* nulls also exhibit an increase in Fbp1 (Fig. 3). Activation of the Fbp1 receptor mobilizes fat energy stores balanced with glycogen utilization (Burmester et al., 2001), especially during periods of heightened energy demands, such as larval wandering and subsequent metamorphosis (Chatterjee and Perrimon, 2021; Merkey et al., 2011). The *dFmr1* nulls presumably increase Fbp1 to better access fat stores, elevate free floating fatty acids and activate mitochondrial β-oxidation (Leboucher et al., 2019). The Fbp1 elevation in *dPHKA2* nulls appears counterintuitive, but we speculate that mobilization of fat body stores becomes the central energy source out of necessity, as in other types of GSD (Kishnani et al., 2019). During larval wandering and subsequent metamorphosis, the buildup of excess fat in the fat body is the primary source for energy (Rehman and Varghese, 2021), which may explain the elevated Fbp1 levels. The shift to fat storage is likely a response to impaired glycogen breakdown (Fig. 2A-C), which is supported by the Fbp1 correction in the DKO animals (Fig. 3). In *Drosophila* metamorphosis, glycogen accumulation is strongly inhibited in favor of *de novo* fat body storage (Chatterjee and Perrimon, 2021; Mattila and Hietakangas, 2017). Consistently, patients with GSD show elevated ketosis levels owing to a compensatory increase in fat oxidation (Kishnani et al., 2019). Note that *dFmr1* may also directly regulate *dPHKA2*, as glycogen metabolism can be hyper-activated via glycogen synthase kinase 3 (*Drosophila shaggy*) OE in the FXS model (Mines and Jope, 2011). Thus, *dFmr1*-*dPHKA2* interactions can occur at multiple levels.

We next focused on neurological consequences of *dFmr1*-*dPHKA2* interactions, particularly functions of NMJs, at which defects are well described for the *Drosophila* FXS model (Song et al., 2022; Zhang et al., 2001). Motor defects characterized in Patient 2 also motivated our investigation at the NMJ, which mediates all the coordinated muscle movement (Bodily et al.; 2001; De Boulle et al., 1993). MitoTracker fluorescence at the NMJ is elevated in both GSD-IX and FXS disease models (Fig. 4). MitoTracker fluorescence measures subcellularly localized mitochondrial function/activity (Harwig et al., 2018; Clutton et al., 2019), indicating the increased metabolic rate at NMJ synaptic terminals. Mitochondrial function responds to changes in fat and glycogen levels due to alterations in the citric acid and β-oxidation cycles (Sriskanthadevan-Pirahas et al., 2022; Cormier et al., 2019). Combining *dPHKA2* and *dFmr1* nulls produces a DKO disease model comparable to the control condition in every metric except mitochondrial activity, which remains elevated in the joint DKO disease model (Fig. 4). The elevated mitochondrial function across mutants compared to the control supports a linked elevated metabolic demand hypothesized for the disease states (Casanova et al., 2023; Wang and McLean, 2022). The similar elevation in mitochondrial function supports the idea that both *dPHKA2* and *dFmr1* act in a common respiratory pathway (Talley and Mohiuddin, 2023), making them prime sites for metabolic disruption. Altered synaptic mitochondrial function may offer insights into the neurological symptoms of Patient 2 (De Boulle et al., 1993), as well as synaptic property changes occurring in the *Drosophila* disease models. However, the lack of detectable ROS changes suggests a compensatory rather than pathological alteration in mitochondrial function, in both single and combined disease models.

As independent disease models, the FXS model manifests strong neurological phenotypes, which the GSD model does not show. Consistently, symptoms of patients with GSD are mild and decrease with age (Smith et al., 2020; Özen, 2007). The *dFmr1* nulls exhibit elevated numbers of both mature and immature NMJ synaptic boutons to generate significantly more overall synaptic contact area (Fig. 5). Greater synaptic arborization with supernumerary boutons is linked to the elevated protein synthesis resulting from the loss of the FMRP translational repressor (Song et al., 2022; Coffee et al., 2010). In human patients and the mouse FXS model, developmentally arrested synaptic connections are a hallmark of the disease state (Davis and Broadie, 2017; Cruz-Martín et al., 2010; Huber et al., 2002). Likewise in the *Drosophila* FXS model, excess satellite or mini-boutons at the NMJ similarly are linked to arrested or delayed bouton development, contributing to synaptic connectivity changes (Siller and Broadie, 2011; Gatto and Broadie, 2008; Pan et al., 2004). Such defects have been directly linked to oxidative stress as well as altered metabolic energy dynamics (Vannelli et al., 2024; Licznerski et al., 2020; Lumaban and Nelson, 2015). We hypothesized that the combined GSD-IX and FXS disease models would exacerbate synaptic defects, but *dPHKA2* loss paradoxically completely restores normal NMJ synapse architecture (Fig. 5). Despite higher mitochondrial activation in DKO animals, this correction suggests a balancing of metabolic energy needs with a strongly compensated synaptogenesis mechanism (Shen et al., 2019; Weisz et al., 2018). This utterly unexpected correction of FXS model synaptic architecture in the combined GSD-IX and FXS comorbidity disease model led to our final studies of NMJ functional neurotransmission interactions in single and double mutant conditions.

The FXS model shows increased NMJ transmission strength, with greater EJC amplitudes (Fig. 6A,B). Consistently, the mouse FXS model manifests circuit-specific elevations in neurotransmission strength, coupled to higher activity-dependent protein synthesis rates (Liu et al., 2022; Contractor et al., 2015; Osterweil et al., 2010; Todd et al., 2003). This heightened synaptic function is linked to greater metabolic demands characterized by increased energy store mobilization (Dionne et al., 2024; Qin et al., 2002). The GDS-IX model shows no neurotransmission changes on its own but, combined with the FXS model, completely rectifies the heightened synaptic function (Fig. 6A,B). Reversal of the FXS neurotransmission elevation suggests reduced metabolic demand, with restored fat and glycogen levels (Andersen et al., 2021). Contrary to our hypothesis, DKO animals paradoxically compensate for FXS synaptic phenotypes. Interestingly, OE of *dPHKA2* phenocopies the *dFmr1* null neurotransmission elevation, which is maintained when both are combined together (Fig. 6C,D). This suggests that *dPHKA2* and *dFmr1* have an epistatic interaction (Pan and Broadie, 2007), where *dPHKA2* elevation may be partially responsible for the *dFmr1* null synaptic transmission increase. Thus, *dPHKA2* loss would compensate for this elevation and restore normal synaptic function, as we have observed (Fig. 6A,B). Similarly, the elevated mEJC frequency and neurotransmission quantal content in the FXS model is rectified by *dPHKA2* co-removal (Fig. 7A-D), but *dPHKA2* OE has no effect on quantal content (Fig. 7E-H). Many of the FXS phenotype reversals observed in the DKO animals could possibly be explained by a reduction in *dPHKA2* levels that might overcompensate for inherent OE in the *dFmr1* null. We will test this mechanism in future interaction studies.

In conclusion, our study demonstrates 100% synthetic lethality in the GSD-IX/FXS double disease model, but fails to explain it. The fact that phenotypes are normalized in DKO animals contradicts our hypothesis of metabolic collapse due to energy deficiency causing synapse impairments. Nevertheless, we still hypothesize that a mismatch between metabolic supply and demand may be causal for the extremely heightened phenotypes in the combined disease state (De Boulle et al., 1993), especially during the highly energy-demanding metamorphosis phase in the *Drosophila* disease model (Merkey et al., 2011). Our current data suggest that DKO mitochondrial changes in larvae are a compensatory mechanism to counteract metabolic challenges, but this does not rule out other effects at different developmental stages, or other metabolic causes. For example, we are currently testing the role of glycogen synthase kinase 3 (*Drosophila shaggy*), which is known to be hyper-activated in the FXS model (Mines and Jope, 2011) and may provide a mechanism for the *dFmr1*-*dPHKA2* metabolic interaction. Regardless of the exact mechanism, the connection between GSD-IX and FXS is apparent in Patient 2 (De Boulle et al., 1993). Our new *Drosophila* disease model confirms this interaction and provides a new platform for human translational studies. Consistent with our results, mammalian FXS models and human clinical data show elevated glycolysis/tricarboxylic acid marker levels and higher mitochondrial function (Vannelli et al., 2024; Licznerski et al., 2020; Weisz et al., 2018), supporting a generalized FMRP-PHKA2 metabolic interaction across species. Our new *Drosophila* disease model provides a means to systematically dissect this mechanism and rapidly test derivative therapeutic interventions to inform human disease research.

## MATERIALS AND METHODS

### *Drosophila* genetics

All *Drosophila* stocks were reared on standard cornmeal/agar/molasses *Drosophila* food at a constant 25°C in humidified incubators with a rotating 12/12-h light/dark cycle. Most of the experiments were performed on males, because the *dPHKA2* gene (*CG7766*) is located on the X chromosome, with the null mutant *dPHKA2^CR00405-TG4.1^*/Y. Females were used in the *dPHKA2* OE experiments. The genetic background control for all studies was *w^1118^*. Most of the lines were obtained from the BDSC (Indiana University, Bloomington, IN, USA). The heteroallelic null *dFmr1* animals were generated by crossing *w^1118^; dFmr1^Δ50M^*/TM6B Tb (BDSC 6930) females to *w^1118^*/Y; *dFmr1^3^*/TM6C Sb Tb males, a kind gift from Tom Jongens (Dockendorff et al., 2002). Hemizygous null *dPHKA2* animals were *w^1118^, CG7766^CR00405-TG4.1^*/Y. DKO animals were generated by crossing *w^1118^, CG7766^CR00405-TG4.1^*/FM7-ChFP; *dFmr1^Δ50M^*/TM6B Tb females to *w^1118^*/Y; *dFmr1^3^*/TM6C Sb Tb males. *dPHKA2* OE was induced by crossing female *w^1118^, CG7766^CR00405-TG4.1^* (Gal4 in the *dPHKA2* locus; BDSC 79254) to male *w^1118^, P{EP}CG7766^G104^* (UAS in the *dPHKA2* locus; BDSC 33442). *dPHKA2* OE in the *dfmr1* null background was done by crossing *w^1118^, CG7766^CR00405-TG4.1^*/FM7-ChFP; *dFmr1^Δ50M^*/TM6B Tb females to *w^1118^, P{EP}CG7766^G104^*/Y; *dFmr1^3^*/TM6C Sb Tb males. All lines were confirmed by western blots and/or reverse transcription quantitative PCR using gene-selective primers. The *Fbp1* RNAi knockdown (Vienna *Drosophila* Resource Center v37881; P{GD5143}) and *Fbp1* OE conditions (FlyORF #F001003; {UAS-Fbp1.ORF.3xHA}) were generated by crossing males to UHI-Gal4 females to drive ubiquitous transgene expression.

### Viability assay

Adults were allowed to lay eggs for 2 h on apple-juice/sugar/agar plates with yeast paste. Plates were incubated at 25°C for 24-26 h, by which time larvae had hatched. For each genotype, 100 hatchling larvae were collected and put into a yeasted, warm cornmeal/yeast/molasses/agar food vial, with the surface of the food well mixed to allow larvae to burrow and feed. Pale brown pupae were counted during metamorphosis in pupal case. Adult flies were sorted and counted after eclosion. Viability for each genotype was calculated from the actual number of animals obtained divided by expected number of animals. To test whether a complex carbohydrate diet alters viability, two special foods were prepared. First, 1 l of water containing 6.2 g *Drosophila* agar (Genesee Scientific) and 18.2 g baker’s yeast (Saf-instant) was boiled and allowed to cool to 50°C before adding 9 g enriched white cornmeal (Sysco), 3 ml propionic acid (Fisher) and 3 g methyl 4-hydroxybenzoate (Thermo Fisher Scientific) in 30 ml 95% ethanol. The mixture was then thoroughly mixed and divided into two equal portions, 73.5 g dextrose (Fisher) or 73.5 g cornstarch (Kroger) was added, and the food was dispensed into fly vials for feeding.

### Morphology assay

Single and double mutant *dPHKA2* genotypes were selected based on the absence of the Tb marker and RFP transgenic label, as well as the presence of GFP fluorescence indicating the presence of the *PHKA2^CRI^* allele. Adults were anesthetized by immersion in 95% ethanol and photographed with a Canon Rebel DLSR camera mounted on a Leica MZ6 dissection microscope. Selected images were processed using Adobe Photoshop contrast and brightness enhancement tools.

### Fat quantification

Fat bodies were imaged in wandering third instars. Selected animals of each genotype were dissected in physiological saline (128 mM NaCl, 2 mM KCl, 4 mM MgCl_2_, 1.0 mM CaCl_2_, 70 mM sucrose and 5 mM HEPES at pH 7.2), with one larva per plate. Internal organs were preserved during the dissection. Intestines and Malpighian tubules were carefully removed after 30 min fixation with 4% paraformaldehyde (EMS, 15714). Photographs were taken with a Canon Rebel DLSR camera mounted on a Leica MZ6 dissection microscope. A buoyancy-based fat quantification assay was performed as previously reported (Hazegh and Reis, 2016), with minor adjustments. Briefly, ten wandering third instars of each genotype were placed into a 50 ml conical vial filled with a solution of 11.5 ml phosphate-buffered saline (PBS) and 9 ml 20% sucrose. Larvae were gently vortexed for 15 s and left to settle for 1 min. The numbers of larvae floating above the conical portion at the tube bottom were counted. The vial was vortexed again, and larvae were counted at the same sucrose concentration a second time. Increments of 1 ml sucrose were then added while repeating previous steps. Two measurements per volume were done for every sucrose concentration and then averaged for every single data point.

### Glycogen quantification

The protocol followed the instructions for the Glycogen Assay Kit (Abcam, ab65620). Briefly, eight wandering third instars of each genotype were homogenized in centrifuge tubes with a glass homogenizer and sonicator in 200 µl ddH_2_0 on ice. Homogenates were boiled for 10 min. In a 96-well black, flat-bottom plate, 1 µl extracted sample and 49 µl loading buffer were placed in reaction wells in duplicate. Sample background controls were similarly duplicated. The colorimetric assay was performed with 2 µl hydrolysis enzyme solution added to standard and sample wells. After 30 min dark incubation, a mixture of 46 µl assay buffer, 2 µl development enzyme mix and 2 µl OxiRed probe was added to each well. Samples were incubated in the dark for a further 30 min. Samples were measured at an optical density (OD) of 570 nm on a POLARstar Omega Platereader (BMG Labtech).

### Western blotting

Four pharate adult (<24 h prior to eclosion) heads per genotype were transferred to standard centrifuge tubes on ice. Heads were homogenized with a handheld motorized homogenizer in 1× NuPage sample buffer (Invitrogen) diluted with ddH_2_0 and supplemented with 5% β-mercaptoethanol. Samples were then sonicated on ice prior to centrifugation at 11,700 ***g*** for 15 min at room temperature (RT). Samples were pelleted, and the supernatant was boiled for 5 min. Extracts were loaded onto 4-15% Mini-PROTEAN TGX Stain-Free Precast Gels (Bio-Rad, 4568083), then electrophoresed and transferred to a nitrocellulose membrane. Samples were run alongside Precision Plus Protein blue pre-stained protein standards (Bio-Rad, 1610373) and transferred with the Trans-Blot Turbo system (Bio-Rad). Blots were then blocked with TBS intercept blocking buffer (Li-COR, 927-60000) for 1 h at RT. Blocked membranes were then incubated with primary antibodies for 16-18 h at 4°C. Primary antibodies used were as follows: anti-Fbp1 (p19, 1:800; Burmester et al., 2001) and anti-α-Tubulin [Developmental Studies Hybridoma Bank (DSHB), G10, 1:10,000]. Membranes were washed with Tris-buffered saline with 0.1% Tween-20 (TBST) 3× for 10 min and then incubated with secondary antibodies for 90 min at RT. Secondary antibodies used were as follows: 700 goat anti-rabbit (Invitrogen, A21038, 1:10,000) and 800 goat anti-mouse (Invitrogen, SA535521, 1:10,000). After washing 3× for 10 min, membranes were imaged using the Li-COR Odyssey CLx system.

### MitoTracker imaging

Wandering third instars were dissected in physiological saline (as above) with one larva of each genotype on a plate. Dissections were labeled with 17.5 µl MitoTracker Orange (Thermo Fisher Scientific, M7510) reconstituted with 1 ml DMSO in 5 ml PBS (Corning, 46-013-CM) for 30 min at RT. Preparations were then washed 3× with PBS for 10 min each. Preparations were then fixed with 4% paraformaldehyde (EMS, 15714) diluted in PBS for 20 min at RT. After washing 3× with PBS for 10 min each, preparations were incubated overnight at 4°C with goat Alexa Fluor 488-conjugated anti-HRP (Jackson ImmunoResearch, 123-545-021, 1:300). Preparations were then washed 3× with PBS for 10 min each, and mounted with Fluoromount G (Electron Microscopy Sciences) onto 25×75×1 mm slides (Fisher Scientific, 12-544-2) with a 22×22-1 coverslip (Thermo Fisher Scientific, 12-542-B). All NMJ imaging was performed using a Zeiss LSM 510 META laser-scanning confocal microscope, with images projected in Zen (Zeiss) and analyzed using ImageJ [National institutes of Health (NIH) open source]. All NMJ intensity measurements were made with HRP signal-delineated *z*-stack areas of maximum projection using ImageJ threshold and wand-tracing tools.

### ROS assay

ROS were assayed as previously reported (Anh et al., 2011). Briefly, wandering third instars were dissected in ice-cold PBS, then incubated in 10 µM 5-(6)-carboxy-2′,7′-dichlorodihydrofluorescein diacetate acetylester (CM-H2DCFDA; Molecular Probes, Invitrogen) for 5 min. Samples were washed 3× for 5 min each in ice-cold PBS. Samples were then fixed in 1% paraformaldehyde for 5 min and washed 3× with PBS. Samples were mounted in cold Vectashield Mounting Medium (Vector Laboratories). Imaging was performed using a Zeiss LSM 510 META confocal microscope, with images projected in Zen (Zeiss) and analyzed using ImageJ (NIH open source). All fluorescence intensity measurements were made on muscle 6/7 NMJ region of interest (ROI) *z*-stacks of maximum projection.

### ATP assay

Measurements were done according to the ATP Assay Kit for highly metabolically active tissues (Abcam, ab83355). Briefly, five wandering third instars of each genotype were collected and homogenized into 100 µl 2 N perchloric acid (PCA) for deproteinization. Samples were kept on ice for 30 min, then centrifuged at 13,000 ***g*** for 2 min, with the supernatant diluted using Assay Buffer XXIII. Excess PCA was precipitated with 2 M KOH until the pH was neutral, and then samples were again centrifuged at 13,000 ***g*** for 15 min. Samples were stored in a −80° freezer overnight. A 96-well Costar Flat Bottom Plate was loaded with two lanes of a standard curve, two lanes of reaction wells, and one lane of background well loaded with 15 µl of sample diluted to 50 µl with Assay Buffer. Trials 1, 2 and 3 were run together, while trials 4 and 5 were run together with the same standard curve. Samples were dark incubated for 30 min, and then measured at an OD of 570 nm on a POLARstar Omega Platereader (BMG Labtech). ATP concentrations were calculated relative to the standard curve, adjusted for the deproteinization dilution factor.

### NMJ structure

Wandering third instars were dissected in physiological saline (as above), fixed in 4% paraformaldehyde in PBS for 10 min and washed 3× with PBS for 10 min each. All subsequent washes and incubations used PBS+0.2% Triton X-100 (PBS-TX). Preparations were incubated overnight at 4°C in mouse anti-DLG (DSHB, 4F3, 1:100), washed 3× with PBS-TX for 10 min each, then incubated for 4 h at RT in Alexa Fluor 488-conjugated goat anti-mouse-IgG (Thermo Fisher Scientific, A-11001, 1:300) and Alexa FluorCy3-conjugated goat anti-HRP (Jackson ImmunoResearch, 123-605-021, 1:300). NMJs were imaged as previously described (Leahy et al., 2023, 2024). Briefly, a 63× oil-immersion Plan Apo objective was used on a Zeiss LSM 510 META microscope. Muscle 4 NMJs in abdominal segment 3 were imaged in *z*-stacks. All images were labeled in a masked manner and analyzed using FIJI software (NIH, open source). Maximum-intensity projections were made using the ‘Z Project’ function. Boutons were defined as swellings along the axon with DLG fluorescence. Using the HRP channel, individual boutons were measured by hand-drawing an ROI around each, to capture its area and the total bouton number in each NMJ. Graphs were prepared and statistical analyses were performed using Prism software (GraphPad Software).

### NMJ electrophysiology

TEVC recordings were collected as reported previously (Leahy et al., 2023, 2024). Briefly, dissections of wandering third instars were performed at 18°C in physiological saline (as above). The staged larvae were dissected longitudinally along the dorsal midline, the intestines were removed, and the body walls were glued down (Vetbond, 3M). Peripheral motor nerves were cut at the base of the ventral nerve cord. Dissected preparations were imaged with a Zeiss 40× water-immersion objective on a Zeiss Axioskop microscope. With two intracellular electrodes (1 mm outer diameter borosilicate capillaries; World Precision Instruments, 1B100F-4) of ∼15 MΩ resistance when filled with 3 M KCl, muscle 6 in abdominal segments 3 to 4 was impaled. An Axoclamp-2B amplifier (Axon Instruments) was used to clamp the muscles at −60 mV. For evoked EJC recordings, the motor nerve was stimulated with a fire-polished glass suction electrode using 0.5 ms suprathreshold voltage stimuli at 0.2 Hz from a Grass S88 stimulator. Nerve stimulation-evoked EJC recordings were filtered at 2 kHz. To quantify EJC amplitudes, ten consecutive traces were averaged, and the average peak value was recorded. Spontaneous mEJC recordings were collected as reported previously (Leahy et al., 2023, 2024). Briefly, 2-min continuous recordings were low-pass filtered at 200 Hz. Quantal content was calculated by dividing evoked EJC amplitude by average mEJC amplitude. Clampex 9.0 was used for all data acquisition, and Clampfit 10.7 was used for all data analyses (Axon Instruments).

### Statistical analyses

All statistics were performed using GraphPad Prism software (version 9.0). Datasets were subject to normality tests, specifically Shapiro–Wilk tests. ROUT outlier tests with Q set to 1% were only used on normal datasets. Normal data were analyzed with a one-way ANOVA followed by Tukey or Sidak's multiple comparison test. Non-normal data sets were analyzed by a Kruskal–Wallis test with a Dunn's multiple comparison test. All figures show all of the individual data points as well as mean±s.e.m. *P*≤0.05 was considered significant.

## Supplementary Material

10.1242/dmm.052183_sup1 Supplementary information
