# Supplementary material for: Interaction between neuromuscular junction metabolic requirements in fragile X syndrome and glycogen storage disease models
Source: Dis Model Mech. 2025 Sep 1;18(8):dmm052183. doi: 10.1242/dmm.052183 (PMC12452063; doi:10.1242/dmm.052183)
Supplement: Supplementary information [file dmm-18-052183-s1.pdf]

Gurijala et al. Figure S1

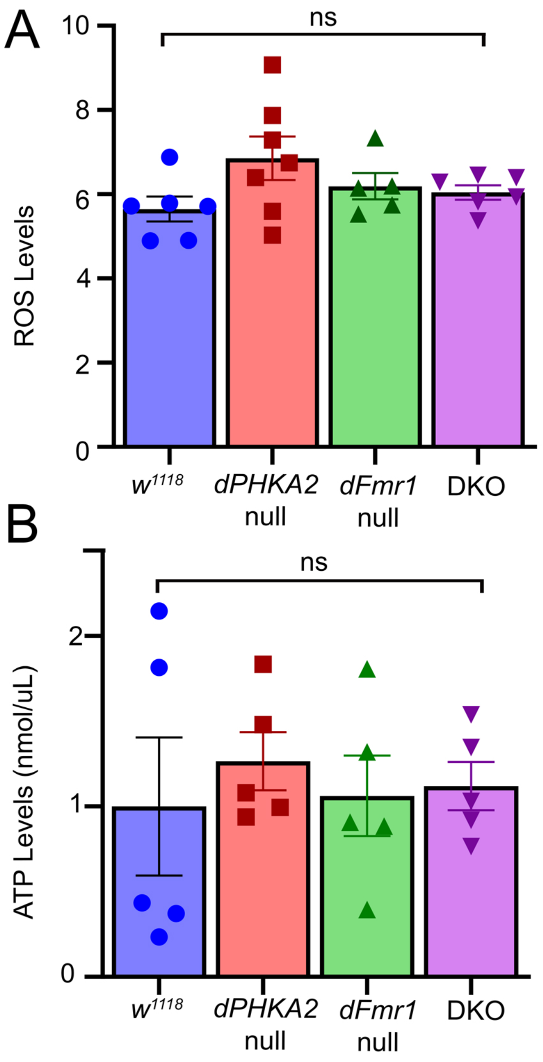

Fig. S1. ROS and ATP levels not detectably altered in single or double mutants. (A) ROS levels in the wandering third instar muscle 6/7 NMJ region of interest (ROI) in genetic background control (w^{1118}), *dPHKA2* null ($dPHKA2^{Cri}$), *dFmr1* null ($dFmr1^{50M/3}$), and the double knockout (DKO). (B) ATP levels in wandering third instars in the same four genotypes. Quantification analyzed using a one-way ANOVA followed by Dunnett's multiple comparisons test. Scatter plots show all data points with mean ± SEM. All comparisons not significant (ns; $p>0.05$).

Gurijala et al. Figure S2

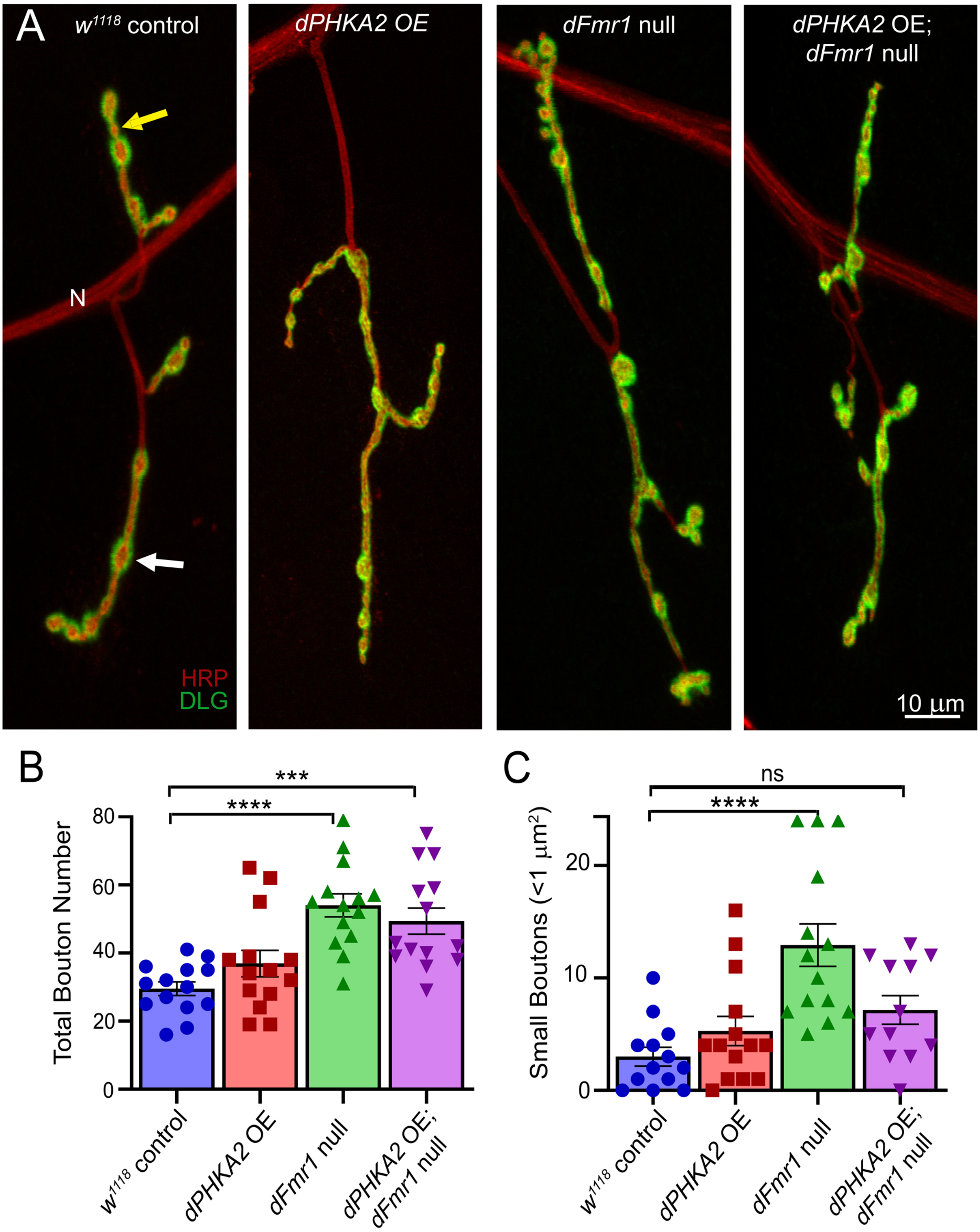

Disease Models & Mechanisms • Supplementary information

Fig. S2. FXS synaptic overgrowth relatively unaffected by *dPHKA2* expression.
(A) Representative confocal images of wandering third instar NMJs co-labeled with presynaptic anti-HRP (red) and postsynaptic anti-Discs Large (DLG, green) to show the synapse structure. Scale bar: 10 μm. Muscle 4 NMJs in the background control (w^{1118}, n=14), *dPHKA2* null ($PHKA2^{Cri}$, n=14), *dFmr1* null ($dFmr1^{50M/3}$, n=14), and the double knockout (DKO, n=14). Scale bar: 10 μm. N indicates nerve, white arrow indicates a mature bouton, yellow arrow indicates a mini bouton. Quantifications of total bouton (B) using one-way ANOVA followed by Dunnett's multiple comparisons test and mini-bouton (C) number using Kruskal-Wallis with Dunn's multiple comparisons test after outliers removed. Scatter plots show all data points with mean ± SEM. Significance indicated as $p \leq 0.0001$ (****), $p \leq 0.001$ (***), and not significant (ns; $p > 0.05$).

Disease Models & Mechanisms • Supplementary information
